# Improving the Quality of Life of Breast Cancer Chemotherapy Patients With Egg Protein Supplementation: Evidence From an Experimental Study

**DOI:** 10.7759/cureus.105415

**Published:** 2026-03-17

**Authors:** Sheikh N Islam, Mahbuba Kawser, Khursheda Akhtar, Mousumi Akter, Md. Saidul Arefin

**Affiliations:** 1 Institute of Nutrition and Food Science, University of Dhaka, Dhaka, BGD; 2 Public Health and Hospital Administration, National Institute of Preventive and Social Medicine, Dhaka, BGD; 3 Nutrition and Food Engineering, Daffodil International University, Birulia, BGD

**Keywords:** breast cancer, chemotherapy patients, egg protein, heath related quality of life, standard

## Abstract

Background: Breast cancer is the most frequently diagnosed cancer in women around the globe; both cancer and its treatment influence breast cancer patients' food intake behaviors and quality of life (QoL). Standard egg protein was supplemented to breast cancer chemotherapy patients, and QoL was assessed.

Methods: A total of 63 breast cancer chemotherapy patients were included in the study. The QoL Questionnaire (EORTC QLQ-30, Version-3) of the European Organization for Research and Treatment of Cancer (EORTC) was used to measure the QoL. The Intervention Group (n=33) received one whole boiled egg and two boiled egg whites per day for 12 weeks, while the Control Group did not. Socioeconomic and cancer-related data, dietary intake, anthropometric measurement (height, weight), biochemical indices (WBC, RBC, platelet count, serum glutamic-pyruvic transaminase (SGPT), serum glutamic-oxaloacetic transaminase (SGOT), serum creatinine, and albumin), and QoL data were collected at three timelines (Baseline, Follow-up at six weeks, and Endline after 12 weeks).

Results: Mean quality of life scores increased from baseline (3.8, 95%CI: 3.5-4.1) to the endline (4.48, 95%CI: 4.3-4.8) in the Intervention Group, while those decreased in the Control Group (n=30) from 4.6 (95%CI: 4.3-4.9) to 3.8 (95%CI: 3.4-3.9). However, weight gain was static (P>0.05) for cases, despite the Control Group losing weight significantly across the three timelines (P=0.03). The biochemical profile revealed that time-dependent increments of lymphocytes, monocytes, eosinophils, and albumin levels occurred only in the Intervention Group (P < 0.05), while insignificant changes were observed in the Control Group across the timelines (P > 0.05). Multivariate analysis of covariance (MANCOVA) revealed that last week's health-activity score (P<0.001), BMI (P=0.003), calorie intake/day (P=0.015), and egg-protein intake/day (P=0.047) were significant time-varying predictors associated with QoL scores. Conversely, non-time-dependent predictors (socioeconomic and cancer-related variables) were found to be insignificant.

Conclusion: Improved QoL scores in the intervention group strongly correlated with the immunonutritional influence of standard egg protein.

## Introduction

Breast cancer was the second leading cancer (after lung cancer) across the globe. In 2022, according to WHO estimates, globally, 2.3 million (11.6%) women were diagnosed with breast cancer, and it was the cause of 670,000 deaths [1]. Breast cancer is the most common cancer in women in the vast majority of countries (157 countries out of 185). It occurs at any age after puberty, but approximately half of them occur with no specific risk factors other than sex and age, and their rates increase in later life [1,2]. The Global Cancer Observatory 2022 reported that the incidence of breast cancer in women in Bangladesh was highest, with 18.0% of all new cancers, while ranked fourth for both sexes (7.8%), and the five-year prevalence of breast cancer was 42.4 per 100000 [3]. 

Breast cancer is a disease in which abnormal breast cells grow (inside the milk ducts or the milk-producing lobules of the breast) out of control and invade nearby breast tissue, creating tumors. Invasive cancers can spread to nearby lymph nodes or other organs (metastasize); metastasis can be life-threatening and fatal [1]. Chemotherapy is one of the modalities to treat patients with breast cancer. However, chemotherapy can induce taste alteration, which leads to poor food intake and nutrient ingestion, increases the risk of lower energy, and consequently induces malnutrition, undermining immunity in chemotherapy cancer patients [4,5], thereby undermining the quality of life (QoL) [6]. 

Global estimates reveal striking inequities in the breast cancer burden. Women in lower human development index (HDI) countries are 50% less likely to be diagnosed with breast cancer than women in high HDI countries, yet they are at a much higher risk of dying of the disease due to late diagnosis and inadequate access to costly, quality treatment. WHO's global survey sheds light on significant inequalities and lack of financial protection for cancer around the world, with populations, especially in lower-income countries, unable to access the basics of cancer care [1,2]. 

Evidence indicates that nutritional issues are considered during cancer diagnosis within a diagnostic and therapeutic pathway and should be parallel to antineoplastic treatments. Cancer-related malnutrition results from many factors, including emotional stress and physical conditions. Malnutrition correlated to infection and suppressed immunity significantly increases discomfort, reduces response and tolerance to treatments, physical functioning, and QoL. Malnutrition also negatively impacts treatment toxicities, and it has been estimated that up to 10-20% of cancer patients die due to the consequences of malnutrition rather than from the tumor itself [7-11]. However, worldwide, cancer-related malnutrition is still vastly unrecognized, underestimated, and undertreated in clinical practice. At present, the five-year survival rate of breast cancer is 86%. Increasing survival rate in breast cancer patients mainly focuses on improving the health-related QoL (HRQoL) through an emphasis on nutrition. It has recently been stressed that the health span is more important than lifespan. Lifespan is the number of years and how long they are alive, while health span is the number of years they could survive with good HRQoL, and how well they live. Thus, nutritional intervention, such as increased protein-rich food, can be an appropriate way to improve health status as well as a better HRQoL in cancer chemotherapy patients [11,12].

Immunonutritional support is one of the most essential parts of anti-cancer therapy [13]. Dietary protein is one of the leading nutrients responsible for building and repairing muscle, tissues, and every cell in the body. Moreover, dietary micronutrients are pivotal in boosting and maintaining immunity [7,13]. An earlier study reported that whey protein supplementation could increase glutathione levels and improve nutritional status and immunity in cancer patients undergoing chemotherapy [14]. Moreover, whey protein's higher essential amino acid leucine content and intrinsic capacity to alter insulin-like growth factor (IGF-I) concentrations were also reported [15]. Egg protein is recognized as the gold standard for lean protein and has the highest attainable protein digestibility-corrected amino acid score, which contributes to energy and immunity, and helps build and repair muscles [14,16]. It provides all of the essential amino acids, lipids (omega-3 fatty acids), minerals (zinc, selenium), and vitamins (B vitamins, vitamins A, D, and E). Moreover, eggs are a good source of choline, high bioavailable carotenoids, lutein, and xanthene, and have protective antioxidants that act against degeneration, fatty liver, and atherosclerosis [16]. Furthermore, eggs contain growth factors and promote protein synthesis, which is required for both growth and development; thus, nutritional supplementation with eggs would be associated with improving HRQoL. Egg white and yolk proteins are functional foods with biological activities, including anticancer and immunomodulatory activities [17-19].

In Bangladesh, the general population has poor knowledge and socially stigmatized attitudes towards breast cancer [20]. Notably, low and middle-income countries (LMICs) like Bangladesh faced unstandardized patient care, high treatment costs for cancer, and the overstretched burden of the resource-poor healthcare system. Cancer care is still inaccessible to hundreds of thousands of cancer patients in Bangladesh due to the lack of a population-based cancer registry (PBCR), late-stage cancer diagnosis, scarcity of trained professionals, unavailability of equipment, and limited access to timely and standard care treatment, leading to higher mortality rates for non-communicable diseases [1,2,20-22]. Furthermore, an intervention study, especially on a standard protein supplementation for breast cancer chemotherapy patients in Bangladesh, is not available in the literature.

Given the importance of having better QoL, disease prognosis, and enhanced treatment compliance in financially poor breast cancer patients who undergo chemotherapy, nutritional status and immunity need to be upgraded through quality nutritional therapy. Gold standard egg protein is easily available, rich in protein (especially leucine), and comparatively cheap and highly bioavailable and digestible for breast cancer patients. Therefore, in this study, cheap and excellent bioavailable standard egg protein was supplemented to breast cancer chemotherapy patients (three eggs daily (one whole, full-boiled egg plus two full-boiled egg whites), and the QoL (primary outcome) was measured by a validated questionnaire, along with both biochemical indices and assessment of dietary intake across three time points and compared with a control group, making the study valuable for all chemotherapy patients in resource-poor healthcare settings in Bangladesh.

Hypothesis statement and outcome variables

The hypothesis of the study is that egg proteins will improve the QoL in breast cancer chemotherapy patients through improving immunonutritional status. The dependent variable (primary outcome) was QoL, and the independent variables (secondary outcomes) were socio-demographics, dietary and nutrient intake patterns, anthropometry (height, weight, BMI), physical activity, and different biochemical markers.

## Materials and methods

This was an experimental study conducted for 12 weeks among breast cancer chemotherapy patients at the National Institute of Cancer Research Hospital (NICRH), Mohakhali, Dhaka, Bangladesh. The objective was to assess whether HRQol was improved by egg protein. The study was approved by the Ethics Committee of the NICRH (approval number: NICRH/Ethics/2020/240, dated November 29, 2020). Both oral and written consent were obtained from the participants, and confidentiality was maintained according to the Helsinki Declaration.

Eligibility criteria

Inclusion criteria were adults aged 18-50 years, with a confirmed diagnosis of breast cancer on stage I or II, who were receiving chemotherapy, and who agreed to participate in the study. Breast cancer patients with cancer on stage III or more, breast cancer patients with comorbidities like chronic kidney disease, cardiovascular diseases, cirrhosis, and patients who did not give written consent or who dropped out of the feedback process were excluded.

Sample size calculation

This study investigated the superiority of the Intervention Group with a Control Group in the absence of reference interventions (Egg supplementation for 12 weeks). Therefore, the classical approach was to frame a two-sided (there is a significant difference in the outcome variable between the two groups) alternative hypothesis. The Consolidated Standard of Reporting Trials (CONSORT) recommends a two-sided P-value and confidence interval approach to declare the statistical significance.

Sample size, $n = \frac{\sigma^{2} \left(1 + \frac{1}{k}\right) \left(Z_{1-\alpha/2} + Z_{1-\beta}\right)^{2}}{(\mu_{1} - \mu_{2})^{2}}$

Here, 

σ^2^ =Expected variability=(6.9)^2^ = 47.61 (SD), (1 + 1/k)= 2 (where, k=1, is the allocation ratio between Control Group and Intervention Group=Arm_1_:Arm_2_=1:1), Z_1- α/ 2_= 1.96 (or .05 level of significance for a 2-sided 95% CI), Z_1-β_ = 1.28 (or 90% power), (Z_1- α/ 2_ + Z_1-β_)^2^=(3.24)^2^=10.4976, µ_1_= 58 (Expected mean outcome or ‘Body weight’ in the Control Group), µ_2_=63 (Expected mean outcome or ‘Body weight’ in the Intervention Group), (µ_1_-µ_2_)^2^=(5.0)^2^ = 25 = Clinically meaningful differences

Thus, the sample size for each group=39.98=40. By adding 10% of dropout cases during follow-up, the estimated sample size was 40+4=44.

Patient recruitment

A computer-generated simple random sampling design was adopted to finally recruit a total of 63 breast cancer chemotherapy patients at stage I or II [23] from the source population of 150 breast cancer patients following the CONSORT guidelines [24], with 33 participants in the Intervention Group and 30 participants in the Control Group. The Intervention Group was supplemented with gold standard eggs and no animal protein/food restriction, while the Control Group had no egg supplementation according to the study protocol. There was no animal protein/food restriction in either group.

**Figure 1:**
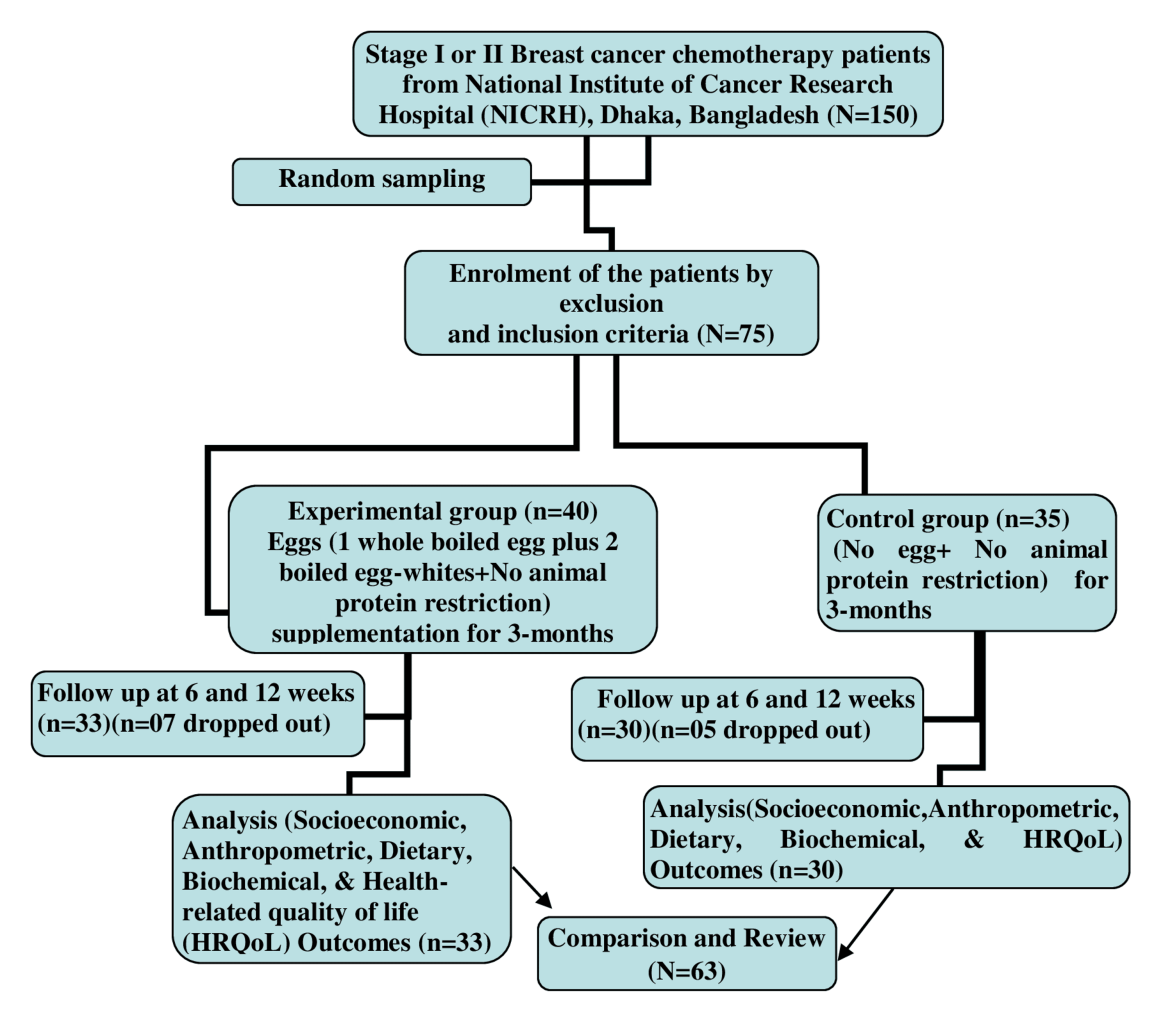
CONSORT diagram of the enrollment, follow-ups, and analytical variables of the study CONSORT: Consolidated Standards of Reporting Trials [24]; HRQoL: health-related quality of life

Intervention

Three eggs (One entire full-boiled egg plus the full-boiled white portion of the other two) were given to the Intervention Group daily for three months, with baseline data collection and the first follow-up (F1) at six weeks and the final follow-up at 12 weeks. All biochemical, dietary intake, and anthropometric data were collected for both groups at three time points (Baseline, F1, and Endline).

Dietary Intake and Supplementation Procedure

Addressing the usual food habits of the breast cancer chemotherapy patients: Before the commencement of the study, both groups were asked about their regular egg and protein intake pattern, which was normal (e.g., taking one egg/day, one glass of milk/day, one piece of chicken/day, and one piece of fish/day), that also echoed with overall food habit and intake per day at the baseline data. Baseline food intake data (data not shown) showed that 53.8%, 73.1%, 80.8% and 96.2% of the breast cancer chemotherapy patients (in both groups) consumed one egg/day, one glass of milk/day, one piece of chicken/day, and one piece of fish per day, respectively. Thus, none of the study participants consumed more than one egg (46.2% did not eat even one egg/day).

Egg supplementation procedure: The Intervention Group was given raw eggs for 10 days (30 eggs/person), and the egg intake pattern per day/person was monitored/confirmed each day over the cell phone for 12 weeks. All patients, including controls, met with the researchers/principal investigator with blood reports 21 days after completion of the chemotherapy.

Animal protein/egg restriction strategy: All the participants, both in the Intervention Group and the Control Group, were patients with breast cancer. The participants had gastrointestinal (GI) symptoms (data not shown) that prevented them from taking food, along with chemotherapy-induced taste alteration. Thus, no restriction was placed on taking any types of food/protein restriction, including egg, while monitoring their food intake pattern over the phone and after completion of the chemo-cycle (after 21 days) [11]. As meat, fish, and eggs are all animal sources of protein and are more bioavailable than plant proteins, we did not restrict the Control Group from having eggs. Moreover, it would be unethical to restrict protein intake, which helps to grow, repair, and maintain the body, for cancer patients. Thus, both groups were allowed to intake their normal/usual foods or other rich/extra protein, such as meat or fish. What the study did was provide two additional boiled egg whites and one boiled whole egg only for the Intervention Group. Thereby, the Intervention Group consumed an extra 108 k calories, 7 g fat, and 14 g protein regularly (on a per-day basis) for 12 weeks as compared to the Control Group. Compliance with the proper egg supplementation in the Intervention Group was checked by taking some steps and providing some advice to the caregivers/guardians of patients of both groups (see Appendix A).

Allocation concealment: As the gold standard egg protein (comprising one boiled whole egg and two boiled egg whites) was supplemented to the Intervention Group, and no placebo was supplied to the Control Group, the study lacked a 'Blind strategy' or 'Allocation concealment' between the two groups.

Assessment tools and data collection procedure

A pretested questionnaire was used to record the data. A skilled researcher and physician collected socioeconomic, cancer-related information, dietary data (24-hour recall at baseline and end line), and anthropometric data (Height and weight measurements at three time points), assisted by a qualified nurse or researcher, in the presence of a physician/principal Investigator (PI).

Blood Collection and Biochemical Indices

A lab technician or paramedic, assisted by nursing staff, collected 7.5 ml of blood aseptically in a heparinized tube. The blood was then processed to analyze various biochemical parameters further, including hematocrit (%), RBC (erythrocytes), WBC (leukocytes), and platelet counts. Additionally, albumin, liver function tests (serum glutamic-pyruvic transaminase (SGPT), serum glutamic-oxaloacetic transaminase (SGOT), and alkaline phosphatase (ALP), and serum creatinine were also assessed. Erythrocytes and leukocytes were evaluated to determine the immunity level (diagnostic procedure of biochemical indices is outlined in Appendix B).

Before chemotherapy for each participant, blood samples were collected routinely to assess the physiological condition of the patients. All laboratory tests were included in their routine tests, rather than taking an extra blood sample.

Conversion of Dietary Intake Data to Macronutrients 

Dietary data (Two 24-hour recalls) of both groups were converted to energy (Kcal), fat (g), total protein (g), egg protein (g), and carbohydrates (g) with the help of the 'Food Conversion Table for Bangladesh' [25].

Calculation of Anthropometric Data

BMI (kg/m²) was calculated by dividing one's weight (kg) by height (m²).

Health-Related Quality of Life

To measure the HRQoL in the intervention and control groups, the QoL Questionnaire (EORTC QLQ-30, version 3) of the European Organization for Research and Treatment of Cancer was used with permission [26]. The EORTC QLQ-C30 (https://qol.eortc.org/) is a self-reporting cancer-specific measure of QoL and includes total 15 scales, including five functional scales assessing physical functioning, role functioning, emotional functioning, cognitive functioning, and social functioning; nine multi- and single-item scales assessing fatigue, nausea and vomiting, pain, dyspnoea, insomnia, appetite loss, constipation, diarrhea, and financial difficulties, and a global health status/QoL scale. Last week’s health activity scores were also measured using 'physical functioning' scales.

Statistical analysis

All sociodemographic, anthropometric, dietary, physical activity, and biochemical data of this study were analyzed using IBM SPSS Statistics for Windows, version 27.0 (IBM Corp., Armonk, New York, United States). The data were cleaned, rechecked, and thoroughly verified to minimize inconsistencies and ensure overall data quality. Cleaned data were checked for normality and collinearity for multivariable analysis. Descriptive statistics were used for both quantitative (mean±SD) and categorical data (number/percentage), and Chi-square tests to estimate categorical differences. For normal continuous data, student’s t-tests or paired sample t-tests were used to assess the mean differences between two groups (Intervention versus Control). Assumptions testing and model specifications were performed using statistical analysis software. Moreover, analysis of covariance (ANCOVA) was conducted to find out the differences within groups across the three timelines (e.g., at baseline, after six weeks, and after 12 weeks). Furthermore, a non-parametric method named ‘Related sample Friedman's two-way ANOVA by Ranks’ was employed mainly to measure the changes of the biochemical Indices (non-normal data) within groups across the three timelines, and the Kruskal-Wallis test for comparing between groups (Intervention Group Vs Control Group). Additionally, advanced statistical methods like multivariate analysis of covariance (MANCOVA) from general linear models (GLMs) were used to estimate the influence of time-varying or non-time predictors/covariates on QoL scores.

A fixed-effect regression model using dummy variables was also used to calculate the coefficient of determination (R^2^) and squared multiple partial R^2^ [27] for time-dependent variables following standard egg protein supplementation for three months in the participants. The analytical technique is outlined in Appendix C. Levene’s test of equality of error variances or homogeneity of covariance matrices was observed for the time-variant dependable variable (QoL). As all sociodemographic and cancer-related data were collected from a random sampling method, and all were categorized, thus minimizing confounding whenever they were used in the analysis, stratification minimized confounding factors. The P-value was set as <0.05 for significance. 

## Results

Sociodemographic and dietary intake characteristics

Table 1 presents the sociodemographic, anthropometric, dietary, and breast cancer-related characteristics of the participants. The mean age of the Intervention Group and Control Group was 42.2 and 47.1 years, respectively (P>0.05). Most of the patients were illiterate (Intervention Group=48.5%, Control Group=53.4%; P>0.05), housewives (Intervention Group=81.8%, Control Group=76.7%; P>0.05), married (Intervention Group=87.9%, Control Group=76.7%; P>0.05), non-smokers (Intervention Group=75.8%, Control Group=80.0%; P>0.05), and had similar mean income (Intervention Group=16,818 BDT, Control Group=15,321; P>0.05). Notably, 66.7% of the Intervention Group were from districts other than Dhaka as compared to the Control Group (40.0%) (P<0.05). Moreover, differences in anthropometric indices between the two groups were noticed as insignificant (P>0.05).

**Table 1:** Sociodemographic, anthropometric, and dietary characteristics of the breast cancer patients ^†^Only five patients completed the Secondary School Certificate (SSC) in the Intervention Group, and four in the Control Group. ^††^Chittagong/Barisal/Mymensingh/Sylhet/Khulna/Rangpur districts in Bangladesh; Dietary intake data were analyzed with the help of  ‘Food Conversion Table’ for Bangladesh [24]; P<0.01^*^; P<0.001^**^

Characteristics		Intervention Group (n=33), n (%)	Control Group (n=30), n (%)	Test-statistics and p-value
Sociodemographics				
Education	Illiterate	16 (48.5)	16 (53.4)	Chi-square test=0.031, P=0.985
	Up to class 5	08 (24.2)	07 (23.3)	
	Secondary (SSC^†^) or above	09 (27.3)	07 (23.3)	
Occupation	Employed	06 (18.2)	07 (23.3)	Chi-square = 1.01, P=0.139
	House-makers	27 (81.8)	23 (76.7)	
Family Income (BDT)	Mean±SD	16,818±1002	15,321±6960	Student' t-test = 2.05, P=0.895
	≤15000	20 (60.6)	19 (63.3)	
	>15000	13 (39.4)	11 (36.7)	
Age (years)	Mean±SD	42.2±8.8	47.1±10.8	t-test =5.05,P=0.035
	≤40	17 (51.5)	10 (33.3)	
	>40	16 (48.5)	20 (66.7)	
Marital status	Married	29 (87.9)	23 (76.7)	Fisher’s exact test=1.92, P=0.125
	Widowed/divorced/separated	04 (12.1)	07 (23.3)	
Residential area	Dhaka	11 (33.3)	18 (60.0)	Chi-square = 3.53, P=0.045
	Outside Dhaka^††^	22 (66.7)	12 (40.0)	
Smoking	Yes	08 (24.2)	06 (20.0)	Chi-square =0.53, P=0.615
	No	25 (75.8)	24 (80.0)	
Baseline Anthropometry				
Height (m)	Mean±SD	1.5±0.1	1.5±0.1	t-test =1.05, P=0.785
	≤1.52	23 (69.7)	14 (46.7)	
	>1.52	10 (30.3)	16 (53.3)	
Weight (kg)	Mean±SD	55.4±9.7	59.4±7.9	t-test =0.095,P=0.905
	≤55	20 (60.6)	08 (26.7)	
	>55	13 (39.4)	22 (73.3)	
BMI (kg/m^2^)	Mean±SD	26.3±4.6	25.4±4.3	t-test =1.00,P=0.445
	≤25.0	18 (54.6)	18 (60.0)	
	>25.0	13 (45.4)	12 (40.0)	
Dietary intake (g/day)				
Total Kcal/day	Baseline, mean±SD	1014.9±164.7	1059.8±203.6	t-test =.459, P=.931
	Endline, mean±SD	1125.5±145.0	1063.0±230.3	t-test =2.95, P=.046^*^
Total carbohydrate	Baseline, mean±SD	118.8±17.9	120.9±19.3	t-test =.435, P=.903
	Endline, mean±SD	120.6±15.3	125.3±20.1	t-test =.832, P=.309
Total protein	Baseline, mean±SD	72.6±27.1	76.2±29.9	t-test =.856, P=.393
	Endline, mean±SD	88.6±26.0	77.1±30.1	t-test =2.99, P=.003^*^
Egg protein	Baseline, mean±SD	5.2±2.0	7.0±1.7	t-test = -3.13, P=.002^*^
	Endline, mean±SD	23.2± 2.3	6.3±1.9	t-test=50.9,P=.000^**^
Total fat	Baseline, mean±SD	17.0±13.4	18.9±13.7	t-test =.796, P=.343
	Endline, mean±SD	22.0±11.4	21.9±12.1	t-test =.126, P=.193

No significant differences were observed in mean nutrient intake between the intervention and control groups at baseline except for the egg protein intake/day, which was found to be higher in the Control Group at baseline (Intervention Group=5.2±2.0 versus Control Group=7.0±1.7 g/day), while significantly higher in the Intervention Group at the endline (23.2±2.3 versus 6.3±1.9 g/ g/day). Similar results were also found (P < 0.05) at the endline in the Intervention Group for total calories (1125.5 ± 145.0 kcal/day versus 1063.0 ± 23.3) and protein intake (88.6 ± 26.0 g/day versus 77.1 ± 30.1) (Table 1).

Breast cancer-related characteristics 

Breast cancer-related characteristics of the study subjects showed that the majority of the intervention and control groups had Invasive ductal carcinoma with the surgical history of modified radical mastectomy (Intervention Group=33.3%, Control Group=90%). Most of the Intervention Group (60.0%) had only one chemotherapy session, while the majority of the Control Group (66.6%) had two to three chemotherapy sessions with neo-adjuvant chemotherapy. Triple negative breast cancer (TNBC) was mostly prevalent in the Control Group (Intervention Group=54.5%, Control group=70.0%). Both groups had similar physical activities (Table 2 ).

**Table 2:** Breast cancer-related characteristics and physical activity of the intervention and ccontrol groups All breast cancer patients were at either stage I or II [23].

Characteristics		Intervention Group (n=33), n (%)	Control Group (n=30), n (%)	Test-statistics and p-value
Type of histopathology	Invasive ductal cell carcinoma	31 (93.8)	30 (100)	Fisher’s exact test=1.20, P=0.455
	Invasive lobular carcinoma	01 (3.10)	00 (0.00)	
	Mucinous carcinoma	01 (3.10)	00 (0.00)	
History of operation	No surgical operation	18 (54.5)	20 (66.7)	Chi-square test=3.53, P=0.04
	Yes	15 (45.5)	10 (33.3)	
Surgical operation (n=15)	Breast-conserving surgery	02 (13.3)	00 (0.00)	Fisher’s exact=0.031, P=0.589
	Modified radical mastectomy	05 (33.3)	09 (90.0)	
	Simple mastectomy	07 (46.7)	00 (0.00)	
	Lumpectomy	01 (6.70)	01 (10.0)	
Chemotherapy number	01	11 (33.3)	20 (66.6)	Fisher’s exact=4.05, P=0.031
	2-3	20 (60.6)	05 (16.7)	
	No	02 (6.10)	05 (16.7)	
Types of chemotherapy	Adjuvant chemotherapy	11 (33.3)	07 (23.3)	Fisher’s exact=2.30, P=0.697
	Neo-adjuvant chemotherapy	21 (63.6)	23 (76.7)	
	Hormone therapy	01 (3.10)	00 (0.00)	
Type of immunohistochemistry	Luminal A	05 (15.1)	01 (3.3)	Fisher’s exact=1.20, P=0.895
	Luminal B	02 (6.20)	02 (6.7)	
	Triple Negative Breast	18 (54.5)	21 (70.0)	
	He-2 rich	08 (24.2)	06 (20.0)	
Physical activity	Yes	19 (30.2)	20 (31.7)	Chi-square=0.023, P=0.569
	No	14 (22.2)	10 (15.9)	

Anthropometric and biochemical indices across the three timepoints 

Table 3 outlines the changes in anthropometric and biochemical indices of the intervention and control groups across three time points (Baseline, six weeks, and 12 weeks). Mean BMI was significantly different between the Intervention Group (26.3±4.6) and the Control Group (25.4±4.3) (P>0.05), while unlike the Intervention Group, the Control Group lost weight during the intervention period (across the three timelines, P<0.05). More importantly, weight gain, BMI, neutrophils, SGPT, SGOT, and creatinine levels among the two groups remained unchanged (P>0.05) across three timelines (within-group differences). Results also showed that the levels of neutrophils, lymphocytes, monocytes, and eosinophils were different between cases and controls (P < 0.05). Moreover, unlike the Control Group, the levels of lymphocytes, monocytes, and eosinophils among the Intervention Group significantly increased from 22.6% to 24.9%, 4.3% to 6.8%, and 2.2% to 3.7%, respectively, across three time points. However, eosinophils increased significantly in the Intervention Group but decreased in the Control Group (P<0.05) across three timepoints. Moreover, total platelet counts were significantly lower in the Control Group; however, the difference was insignificant across the three time points between groups (P > 0.05). Notably, albumin levels increased dramatically in the Intervention Group (three times), while no significant difference was observed between the groups in mean albumin level after the intervention.

**Table 3:** Changes in anthropometric and biochemical indices of the intervention and control group across the three timepoints Analysis of Covariance (ANCOVA) and Paired sample t-test (Parametric); F-ANOVA=Related-samples Friedman's Two-Way Analysis of Variance by Ranks (non-parametric); K-Wallis= Kruskal-Wallis Rank test (non-parametric); Significant= ^*^P<0.05 HCT: hemocrit; SGPT: serum glutamic pyruvic transaminase; SGOT: serum glutamic-oxaloacetic transaminase

Cancer-related Indices	Intervention group (n=33), mean±SD			ANCOVA or related-samples Friedman's 2-way ANOVA by ranks, P-values within Intervention Group	Control group (n=30), mean±SD)			ANCOVA or Related-samples Friedman's 2-way ANOVA by ranks, P-values within Control Group	Paired sample t-test or Kruskal-Wallis rank test, P-values between Intervention Group and Control Group
	Baseline	Follow-up 1	Follow-up 2		Baseline	Follow-up 1	Follow-up 2		
Anthropometry									
BMI (kg/m^2^)	26.3±4.6	26.5±4.6	26.7±6.8	ANCOVA=1.678, P=0.75	25.4±4.3	24.1±4.5	23.5±3.2	ANCOVA=4.76, ^*^P=0.04	t-test=3.5, ^*^P=0.03
Weight (kg)	55.4±9.7	55.5±9.5	55.8±8.5	ANCOVA=1.865, P=0.35	59.4±7.9	57.8±8.9	56.9±8.7	ANCOVA=6.43, ^*^P=0.03	t-test=5.5, ^*^P=0.02
Bio-chemical									
Total WBC count	7.5±1.9	6.9±6.0	5.90± 2.3	F-ANOVA=9.10,^*^P=0.02	8.7±2.3	8.8±3.7	8.5±8.2	F-ANOVA=7.04, ^*^P=0.03	K-Wallis=0.3,P=0.15
Neutrophils (%)	69.0±10.9	67.4±18.2	65.2±18.3	F-ANOVA=1.125, P=0.345	72.6±38.5	73.8±16.9	72.7±20	F-ANOVA=1.05, P=0.45	K-Wallis=3.12, ^*^P=0.04
Lymphocytes (%)	22.6±12.9	24.4±9.2	24.9±14.6	F-ANOVA=5.45, ^*^=0.04	20.8±6.5	17.9±12.2	19.2±1.6	F-ANOVA=1.94, P=0.56	K-Wallis=4.51, ^*^P=0.04
Monocytes (%)	4.3±2.7	6.7±5.2	6.8±4.7	F-ANOVA=4.13, ^*^P=0.03	3.4±1.4	4.9±4.2	4.9±2.7	F-ANOVA=0.34, P=0.25	K-Wallis=1.40, P=0.20
Eosinophil (%)	2.2±2.1	3.2±4.2	3.7±3.5	F-ANOVA=6.04, ^*^P=0.04	3.1±4.9	3.0±4.8	2.8±3.1	F-ANOVA=5.45, P=0.02	K-Wallis=5.32,^*^P=0.04
Total RBC count	4.9±5.1	4.1±0.8	3.9±0.4	F-ANOVA=4.345, ^*^P=0.04	4.3±0.5	4.2±0.7	4.1±0.9	F-ANOVATS=3.1,^*^P=0.04	K-Wallis=0.39, P=0.90
Total Platelet count	346.8±111	345±123	315.9±109	F-ANOVA=1.435, P=1.05	335.8±79	316.2±136	342.4±155	F-ANOVA=1.0, P=0.35	K-Wallis=3.30, ^*^P=0.04
HCT (%)	36.8±10.4	35.7±6.9	33.8±3.4	F-ANOVA=5.345, ^*^P=0.03	35.9±3.7	35.3±5.7	34.8±7.7	F-ANOVA=5.92, ^*^P=0.02	K-Wallis=0.50, P=0.17
Serum creatinine (mg/dL)	0.62±0.10	0.60±0.14	0.6±0.1	F-ANOVA=2.00, P=0.09	0.6±0.2	0.62±0.2	0.7±0.2	F-ANOVA =1.4, P=0.65	K-Wallis=0.24, P=0.23
Serum bilirubin (mg/dL)	0.46±0.16	0.45±0.21	0.47±0.15	F-ANOVA=1.95, P=0.95	0.39±0.13	0.41±.11	0.48±0.15	F-ANOVA=6.35, ^*^P=0.03	K-Wallis=4.41, ^*^P=0.04
SGPT(U/L)	30.8±19.2	42.5±39.2	44.5±44.4	F-ANOVA=1.30, P=0.45	34.1±40.2	32.7±26.0	32.6±21.0	F-ANOVA=4.09, ^*^P=0.03	K-Wallis=1.30, P=0.32
SGOT(U/L)	35.2±22.0	44.9±23.8	43.6±15.8	F-ANOVA=1.35, P=0.90	36.5±28.7	41.6±20.0	44.1±14.2	F-ANOVA=4.35, ^*^P=0.04	K-Wallis=1.15, P=0.15
Serum Alkaline phosphatase (U/L)	220.2±85	231.6±126	300.0±106	F-ANOVA=5.51, ^*^P=0.02	170.7±11	271.1±180	288.3±155	F-ANOVA=6.11, ^*^P=0.02	K-Wallis=4.34,^*^P=0.03
Albumin (g/dl)	3.9±0.6	4.3±0.7	4.4±0.3	F-ANOVA=4.95, ^*^P=0.019	4.3±0.8	4.4±0.9	4.4±0.7	F-ANOVA=3.99, ^*I^P=0.01	K-Wallis=2.12, P=0.95

QoL and last week's health-activity scores

Table 4 presents the QoL scores for the intervention and control groups across the three timelines (P<0.001). Last week's health-activity scores (4.36 versus 4.0) between intervention and control groups across three timelines also showed a significant difference. The differences among baseline, follow-up 1, and endline (within timeline differences) also varied both in the intervention and control groups. Thus, the grand mean QoL score for the Intervention Group (4.33, 95% CI 4.29-4.71) was higher than that for the Control Group (4.10, 95% CI 3.9-4.30). Similarly, grand health scores for the Intervention Group (4.36, 95% CI 4.19-4.56) were significantly higher than the control group (4.00, 95% CI 3.88-4.26).

**Table 4:** Quality of life (QoL) and health-activity scores of the breast cancer patients across the three timelines The European Organization for Research and Treatment of Cancer Questionnaire (EORTC QLQ-C30) was used for the assessment of Quality of Life (QoL) scores [26] with permission ^*^Analysis of covariance (ANCOVA); ^**^Paired-sample t-test; ^***^Significant=P<0.001

Quality of Life	Intervention group (n=33)			Control group (n=30)			P-values, between-group effects
	Baseline	Follow-up1	Follow- up2	Baseline	Follow-up1	Follow-up2	
QoL score	3.82±0.74	4.73±0.52	4.48±0.79	4.63±0.72	3.93±0.69	3.8±0.71	^*^F (1,61)=17.68, ^***^P<0.001
95% CI	3.5-4.1	4.5-5.0	4.3-4.8	4.3-4.9	3.7-4.2	3.4-3.9	
QoL score (Grand mean)	-	4.33	-	-	4.10	-	^**^t-test=7.89, ^***^P<0.001
95% CI	-	4.29-4.71	-	-	3.9-4.30	-	
Last week’s Health Activity score	4.0±0.8	4.6±0.7	4.5±0.8	4.3±0.9	4.0±0.6	3.8±0.7	^*^F (12,50)=15.97, ^***^P<0.001
95% CI	3.8-4.2	4.0-4.8	4.0-5.0	4.0-4.9	3.5-4.5	3.6-4.2	
Health score (Grand mean)	-	4.36	-	-	4.00	-	^**^t-test=7.10, ^ ***^P<0.001
95% CI	-	4.19-4.56	-	-	3.88-4.26	-	

Predictors of QoL scores

In Table 5, the interaction effect showed that different time-varying independent variables/IVs like last week’s health activity scores, BMI, egg protein intake/day, and total kcal intake/day were observed to be significant on time-dependent QoL scores of the included patients. The interaction indicates that the variation in the means on QoL over the repeated measurement occasions varies as a function of group (cases versus control) membership. However, carbohydrate intake could not reach a significant level. No interaction effect between socioeconomic status, physical activity, and cancer-related variables on QoL scores was observed. The effects of time-non-varying predictors (e.g., all socioeconomic and cancer-related independent variables/IVs) do not depend on QoL.

**Table 5:** Factors influencing the quality of life (QoL) in breast cancer chemotherapy patients the across the three-timelines ^*^Multivariate analysis of covariance (MANCOVA); R^2^=Co-efficient of determination; ^**^Cohen, 1988 [27] for the formula for squared multiple partial R^2^, and about  61.4% (R^2^=.614) variation accounted for by these time-varying predictors, P=.01.

Predictor types	^*^F-values	^*^P-values	^*^Effect size (Partial eta squared)	Fixed effect regression model using dummy variables
Time-varying predictors				
Last week's Health activity score (all three timelines)	F(4, 58)=32.4	P=.000	.694-.848	(1) R^2^=.435
End line Egg protein intake	F(4, 58)=4.10	P=.047	.066	(Between-group effect in model-1)
End line total Kilo calorie /Kcal	F(3, 59)=6.29	P=.015	.096	(2) R^2 ^=.650
Endline total carbohydrate intake	F(3, 59)=4.51	P=.08	.071	(Between-group effect +time-varying predictors effect in model-2)
Endline BMI (kg/m^2^)	F(4, 58)=10.34	P=.003	.151	(3) ^ **^Squared multiple partial R^2^=.614
Time-In varying predictors				
Marital status (married/widowed/divorced/separated)	F(13, 49)=4.62	P=.055	.074	No effect
Smoking (Smokers/non-smokers)	F(13, 49)=3.34	P=.074	.064	No effect

**Figure 2:**
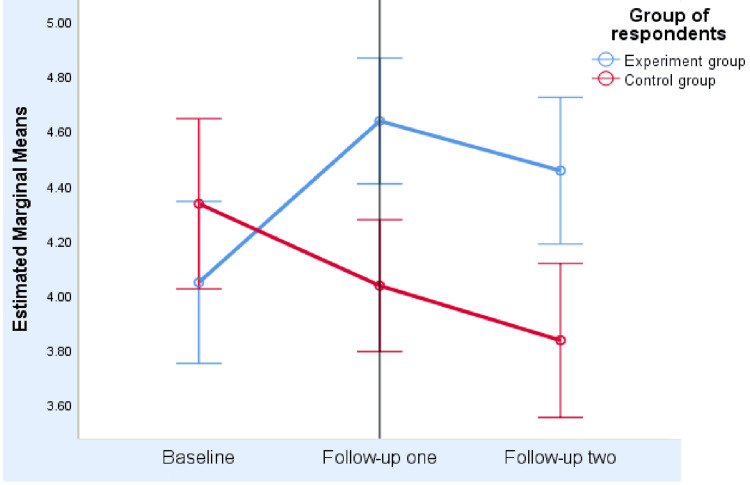
Quality of life (QoL) scores between intervention (experiment) and control groups across the three timelines for the breast cancer chemotherapy patients The European Organization for Research and Treatment of Cancer Questionnaire (EORTC QLQ-C30) was used for the assessment of Quality of Life (QoL) scores with permission [26]. In this profile plot, QoL scores of the Intervention Group significantly increased as compared to the Control Group across the three timelines.

In Figure 2 (profile plot), the QoL scores of the Intervention Group were significantly increased from baseline to follow-up 2 (endline) as compared to the Control Group. The profile plot clearly shows that the lines are not parallel. However, the variations in each group across the timelines within all time-variant groups were not linear but quadratic.

## Discussion

Breast cancer is the most frequently diagnosed cancer in women around all regions of the world, although the five-year survival rate is increasing because of immense emphasis on nutrition intervention and HRQoL. This intervention study showed a significant improvement in the QoL in breast cancer chemotherapy patients who consumed a supplementary one whole boiled egg and two boiled egg whites daily for three months, in addition to the regular daily dietary intake or animal/plant protein consumption. Moreover, the biochemical profile of the patients outlined that lymphocytes, monocytes, and eosinophils levels were higher in the Intervention Group (P<0.05) than in the Control Group. In contrast, neutrophils were found to be lower in the Intervention Group than in the Control Group (P<0.05). Furthermore, a decrement of lymphocytes in control across three timelines (within-group differences) was also observed (P<0.05), which may be correlated to the immune-nutritional influence of standard egg protein [13,16-18]. 

A study by Lee and Paik (2019) outlined that the highly bioavailable protein and peptide contents of eggs are responsible for the underlying mechanism of anticancer and immunomodulatory activities [18]. This bioavailable protein and peptides (having a smaller number of amino acids than protein) have been reported to induce apoptosis in cancer cells, protect against DNA damage, and help to decrease the invasion ability of cancer cells, and exhibit cytotoxic and anti-mutagenic activity in various cancer cell lines. 

Lee et al.(2022) showed that when an egg yolk protein sequentially hydrolyzed by two enzymes, pancreatin and neutrase (EYPH-PN), it could enhance the production of tumor necrosis factor (TNF) and interleukin (IL)-6 at both the mRNA and protein levels in macrophages, in addition to increasing the phagocytic activity of macrophages [19].

The dietary analysis in this study reported that at baseline, daily total energy (1059.8 kcal/day), egg protein intake (7.0 g/day), and total protein intake (76.2 g/day) in the Control Group were higher (P>0.05) than in the Intervention Group (1014.9 kcal/day; 5.2 g/day; 72.6 g/day, respectively). However, after a three-month intervention period, these calories and nutrients significantly increased only in the Intervention Group (1125 kcal/day; egg protein 23.2 g/day, total protein 88.6 g/day), although it was far from the nutrient adequacy or intake amount of cancer patients in Portugal reported by Santos and colleagues (2020) [10]. Such increments in energy and protein intake in the Intervention Group were also observed in another study after whey-protein supplementation in breast cancer patients [14]. In contrast, in a recent prospective study by Schmidt et al. (2023) that aimed to find out the associations of prostate cancer risk and mortality with dietary intakes of total protein and amino acids from different dietary sources, the findings elucidated no association with egg protein intake and prostate cancer mortality risk, while a weak positive association for dairy and yogurt protein was found for prostate cancer risk or mortality [28]. Thus, a large-scale study is needed. 

Notably, the body weight of the Intervention Group was static (P>0.05) within the three timelines, while the Control Group lost more weight (P<0.05). A recent review outlined that the prognostic impact of weight loss on overall survival has long been recognized. Data suggested that even a little weight loss (2.4%) predicts survival independent of disease, site, stage, or performance score [29]. These effects may be the influence of three months of highly bioavailable, protein (higher amount of leucine) rich egg supplementation, which may bring on chemotherapy-induced taste alteration to maintain a constant body weight and serum albumin levels only in the Intervention Group [4,5,16-18]. Layman and Rodriguez (2009) showed that increased tissue levels of leucine combine with circulating insulin to allow skeletal muscles to manage protein metabolism and fuel selection in relation to diet composition [16]. Moreover, muscle recovery from exercise, both resistance and endurance, seems to be dependent on dietary leucine. However, a recent meta-analysis reported that dietary intervention did not modify weight, BMI, and hip circumference [30].

Multivariate advanced analysis (Table 5) showed that time-varying predictors like last-week health activity scores (large effects for all timelines), endline egg-protein intake and endline total calorie intake (medium effect for both), and endline BMI (large effect) were significantly associated with improved time-dependent QoL scores, derived from Jacob Cohen's (1988) formula of squared multiple partial R-square [27], while most of the non-time-dependent predictors like socioeconomic and cancer-related variables, and physical activity were not associated with QoL scores. The findings of a recent study by Mistry et al. align with this and outlined that patients with low BMI (<18.5 kg/m^2^) exhibited significantly poorer QoL [31]. A healthy lifestyle and physical activity have been associated with a better quality of life, a good prognosis, and lower mortality.

This study attempted to improve the QoL of breast cancer chemotherapy patients with egg supplementation, which may have helped to alter chemotherapy-induced taste degeneration and increase the food/energy intake or nutrient ingestion. Özkan et al.* *(2022) reported that the chemotherapy-induced taste alteration scale (CiTAS) score was found to be higher in malnourished patients than in those with normal nutritional status [4]. Additionally, eggs are a highly nutritious food that provides specific health benefits for humans. It contains all proteins (especially higher amounts of leucine), lipids, vitamins, minerals, and growth factors necessary for embryonic development. Egg white and yolk are considered functional food substances as they possess biological activities, including anticancer and immunomodulatory activities [13,15-19]. On top of that, egg supplementation for three months significantly improves QoL among cancer patients, and egg protein itself showed a positive medium effect on QoL among breast cancer patients.

Strengths and limitations of the study

One of the primary strengths of this study is the use of a validated and widely recognized EORTC-QLQ-3C0 questionnaire. Moreover, the breast cancer chemotherapy patients had no metastasis or recurrence, and both groups had similar socioeconomic characteristics. Also, measurement of different secondary outcomes (biochemical characteristics, anthropometry, and dietary intake) for both groups across the three time points also strengthens the study. On top of that, advanced statistical analysis revealed a large effect size of the time-dependent predictors (especially end-line total calorie intake, egg protein intake, and end-line increased BMI) influencing the QoL of the breast cancer patients who underwent cytotoxic chemotherapy cycles. The intervention is practical and has potential impact on low-resource settings, and findings of this study from an immunonutritional standpoint for a resource-poor health infrastructure and high-priced cancer care settings would benefit all chemotherapy patients.

However, being a single-centered study with a small sample size may cause an increased probability of a false negative finding in the control arm. Additionally, the application of a non-restrictive phenomenon for any animal protein intake (including egg) for both groups may be considered a limitation. Future studies employing longitudinal designs and multi-institutional samples with targeted intervention strategies are recommended.

## Conclusions

This study showed that egg supplementation significantly improved QoL in breast cancer patients undergoing chemotherapy, as assessed by the validated EORTC-QLQ-30, indicating improved mobility and health status, as well as better immunonutritional status. A three-month standard egg protein supplementation strategy demonstrated that QoL can be improved substantially with an increase in dietary egg protein intake, along with increases in leukocyte (WBC) markers such as lymphocytes, monocytes, and eosinophils, thereby improving immunonutritional status. This nutritional support during chemotherapy can resolve adverse negative side effects of chemotherapy (e.g., nausea, vomiting, anorexia, and impaired taste perception) and optimize treatment tolerance. Such interventions could enhance treatment tolerance, improve nutritional status, and ultimately contribute to better clinical outcomes through improved QoL in all chemotherapy patients. Additionally, this study filled a critical gap in the literature of global cancer care strategies, providing experimental evidence of low-cost and standard protein supplements for resource-poor, overstretched, and costly healthcare settings of LMICs like Bangladesh.

High standard egg supplementation to breast cancer chemotherapy patients can enhance the QoL of all cancer patients through decreasing the possibilities of low food/nutrient/energy intake. Thereby, betterment of health and nutritional status, immune-enhancing function, and anticancer activities can be maintained. Daily egg protein intake consequently increased the total calorie and protein intake/day, increased health scores, and BMI (>18.5 kg/m^2^). In summary, egg protein supplementation significantly increased the QoL scores of breast cancer chemotherapy patients from baseline to the end line of the study. 

## Appendices

Appendix A

Compliance with the proper egg supplementation was checked by taking the following steps: 

(1) Responsible family member/caregiver for each of the patient was selected before study commencement, who took care of the patient for the three-month study period, (2) Responsible caregivers were asked over mobile phone every day and continued it up to the last day (12 weeks of study period), (3) Patients were supplied a brochure that described the proper intake of food, benefits of protein intake, and why the patient should eat at least one egg per day, (4) All patients were also advised to take one bowl pulses in each meal, one handful nut daily (8-10 pieces), one glass of milk at night daily, fresh vegetables with garlic and ginger, as both have anticancer properties, and also to do some physical activity daily, (5) All patients were forbidden to take red meat or processed meat/foods, (6) Protein in main menu should contain three pieces of meat/fish in lunch and in dinner each.

Appendix B

**Table 6:** Standard biochemical analytical techniques employed in the study

Profile	Biochemical Tests	Reference values of the biochemical parameters	Machine	Method
Routine blood tests	RBC count, WBC count, Platelet count, and Hematocrit (%)	RBC:4.3±0.5 X 10^6^/ µl; WBC:4-11 X 10^3^/ µl; Platelet: 150-450 X 10^3^/ µl; Hematocrit (%): 37-47	Hematology analyzer-Sysmex	Automated cell counter
Liver function test	Aspartate aminotransferase (AST/SGOT), Alanine aminotransferase (ALT/SGPT), Alkaline phosphatase (ALP), and Serum Bilirubin (mg/dl).	AST: Normal up to 37 U/L; ALT: Normal up to 41 U/L; Alkaline phosphatase: 98-279 U/L; Serum Bilirubin: <1.2 mg/dl	Selectra ProM	Automated biochemistry analyzer: Kinetic method
Kidney function test	Serum Creatinine (mg/dl).	Serum Creatinine: <1.2 mg/dl	Selectra ProM	Automated biochemistry analyzer: Kinetic method
Protein status	Albumin (g/dl).	Albumin-3.8-5.1 g/dl	Selectra ProM	Automated biochemistry analyzer: Enzymatic method

Appendix C

Data structure for “Fixed-Effect Regression Model using dummy variables": (1) All cases (or IDs) where treatment groups (n=33), and control groups (n=30), were converted to a ‘Long format’ along with three timelines (thus, the total number of cases was 189, intervention group was 99, control group was 90), as each case tripled, representing three timelines). (2) Estimation of dummy variables for “Group” (treatment and control), “Time” (baseline, follow-up at three weeks, and end line at 12 weeks), time-varying dependable variable/DV (QoL), time-varying predictors (BMI, intake of egg protein, total protein, total calorie, total carbs and fat), and non-time-dependent different predictors (socioeconomic, cancer-related variables, physical activity), keeping the first one as “Reference category” for all models. (3) Hierarchical linear regressions were performed twice, where QoL was always the dependent variable: first, for “Model-1”, incorporating “Between-subject” variables like the dummy treatment group itself, all cases except for the first one, and second, for “Model-2”, incorporating all time-varying predictors.

**Table 7:** Squared multiple Partial R-square (R2) enumeration of time-varying predictors for predicting factors influencing the quality of life (QoL) in breast cancer patients across the three timelines R^2^=Coefficient of determination; (4)Jacob Cohen’s (1988) formula for the Squared multiple partial R-squared  (R^2^) [26].

Predictor types	Statistical procedure and formula
Time dependent	
Last week's Health activity score (all three timelines)	Multivariate analysis of covariance (MANCOVA) and “Fixed effect regression model using dummy variables” from General Linear Model (GLM).
End line Egg protein intake	(1) R_1_-square change=.435 (Between-group effects in model-1), F=6.1, P=.013 (Model-1)
End line total kilocalorie (Kcal)	(2) R_2_-square change=.650 (Between group effects in model-1+Time-varying predictors effect in model-2), F=119.8, P=.000 (Model-2)
End-line total carbohydrate intake	(3) Squared multiple partial R^2^=.614=61.4% variation accounted for by Time-varying predictors.
End line BMI (kg/m^2^)	(4) Squared multiple partial R^2^ formula = (R_2_-R_1 _squared changes)/(1-R_2_ square change im model2)
Non-time dependent variables	
Marital status (married/Widowed/divorced/separated)	Multivariate analysis of covariance (MANCOVA) from GLM (Multivariate) was performed (i.e., Regression + ANCOVA) to see the effect of socioeconomic and cancer-related variables (Covariate/Independent variables/IVs) on Time-Varying QoL (Dependent variable/DV)
Smoking (Smokers/non-smokers)	
